# Inflammatory Signalling Associated with Brain Dead Organ Donation: From Brain Injury to Brain Stem Death and Posttransplant Ischaemia Reperfusion Injury

**DOI:** 10.1155/2013/521369

**Published:** 2013-04-15

**Authors:** Ryan P. Watts, Ogilvie Thom, John F. Fraser

**Affiliations:** ^1^Critical Care Research Group, The Prince Charles Hospital, Rode Road, Chermside, QLD, Australia; ^2^Department of Emergency Medicine, Princess Alexandra Hospital, 199 Ipswich Road, Woolloongabba, QLD, Australia

## Abstract

Brain death is associated with dramatic and serious pathophysiologic changes that adversely affect both the quantity and quality of organs available for transplant. To fully optimise the donor pool necessitates a more complete understanding of the underlying pathophysiology of organ dysfunction associated with transplantation. These injurious processes are initially triggered by catastrophic brain injury and are further enhanced during both brain death and graft transplantation. The activated inflammatory systems then contribute to graft dysfunction in the recipient. Inflammatory mediators drive this process in concert with the innate and adaptive immune systems. Activation of deleterious immunological pathways in organ grafts occurs, priming them for further inflammation after engraftment. Finally, posttransplantation ischaemia reperfusion injury leads to further generation of inflammatory mediators and consequent activation of the recipient's immune system. Ongoing research has identified key mediators that contribute to the inflammatory milieu inherent in brain dead organ donation. This has seen the development of novel therapies that directly target the inflammatory cascade.

## 1. Introduction

Organ transplantation is the gold standard treatment for patients with end stage solid organ failure. An ever increasing disparity between available organs and potential recipients is the cause of avoidable morbidity and mortality [1–4]. Ongoing efforts are being made to increase the quantity and quality of organs available for transplant. Although outcomes from non-heart-beating donors have become increasingly successful [5], the majority of organs are still donated from donors after brain death (BD). Significant brain injury of any aetiology will cause a systemic response [6], creating a proinflammatory environment prior to the occurrence of brain death itself. BD then also creates a variety of inflammatory, haemodynamic and endocrine effects, which induce adverse sequelae in distant organs [7–10]. Finally, ischaemia-reperfusion injury (IRI), inherent in transplantation, generates reactive oxygen species (ROS), activates complement, and independently drives cytokine release and inflammation [11, 12]. Every transplanted organ from a BD donor will face these stages of potential injury. Consequently, donor management must consider each step from donor to recipient in order to maximise recipient outcomes. The purpose of this paper is to explore the current understanding of the three main contributors to injury that an organ will travel through from donor to recipient. Additionally, donor management and organ preservation strategies that are currently being investigated will be briefly considered.

## 2. Stage Zero of Potential Organ Injury: Current Concepts in Immunological Signalling

**Figure 1:**
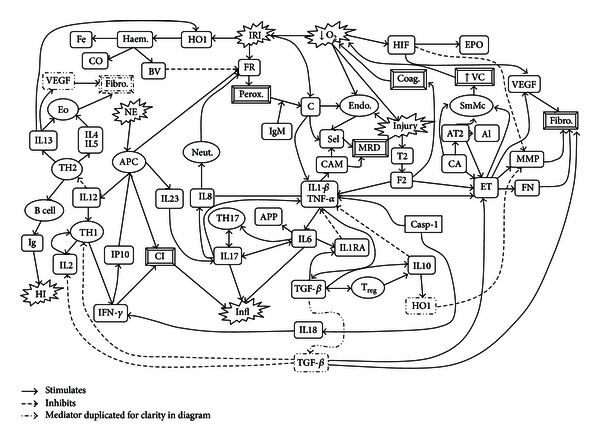
Primary mediators of peri-transplant related inflammation. Al: aldosterone, APC: antigen presenting cell, APP: acute phase proteins, AT2: angiotensin II, BV: biliverdin, C: complement, CA: catecholamines, CAM: cellular adhesion molecule, Casp-1: caspase 1, CI: cellular inflammation, CO: carbon monoxide, Coag: coagulation, Endo: endothelial cells, Eo: eosinophils, EPO: erythropoietin, ET: endothelin, F2: factor II (Thrombin), Fe: iron, Fibro: fibrosis, FN: fibronectin, FR: free radicals, HI: humoral immunity, HIF: hypoxia inducible factor, HO1: heme oxygenase 1, IFN: interferon, Ig: immunoglobulin, IL: interleukin, IL1RA: interleukin 1 receptor antagonist, Infl: inflammation, IP: interferon-*γ*-induced protein, IRI: ischaemia reperfusion injury, MMP: matrixmetalloproteinases, MRD: margination/rolling/diapedesis, NE: new antigens/neoepitopes, Neut: neutrophils, O_2_: oxygen, Perox: peroxidation, Sel: selectin, SmMc: smooth muscle contraction, TF: tissue factor, TGF: transforming growth factor, TH1: type 1 helper T-cell, TH17: type 17 helper T-cell, TH2: type 2 helper T-cell, TNF: tumour necrosis factor, Treg: regulatory T-cell, VC: vasoconstriction, VEGF: vascular endothelial growth factor.

Inflammation, secondary to brain injury, BD, and IRI, is driven by both the innate and adaptive immune systems. The complexity of these systems means that our understanding continues to evolve at a rapid pace (Figure 1). Prior to reviewing the specific inflammatory responses at each major step of the donor organ journey, it is important to discuss current concepts in the normally functioning immune system. 

Traditionally, T-cell responses are grouped according to the T_H_1/T_H_2 paradigm. T_H_1 lymphocytes (CD4^+^) are responsible for cell-mediated immunity through activation of killer CD8^+^ T cells and cytotoxic macrophages [13, 14]. T_H_2 cells are responsible for the control of humoral immunity through antibody producing B cells. Additionally, they regulate eosinophil and basophil functions. Recent work has identified T_H_17 and T-regulatory (T_reg_) subsets. T_H_17 cells have been implicated in autoimmunity [13, 14]. T_reg_ cells are related to T_H_17 cells and function to regulate immunological reactions and prevent uncontrolled inflammation. Each of these T cells plays a specific role in inflammation and their actions can be identified by certain inflammatory mediators. Although cytokines may interact with multiple T-cell subsets, previous authors have classified the major cytokines into “types” reflecting the major T-cell subtype to which they are related [15–18]. This convention will be used in the current paper.

### 2.1. T_H_1-Cell-Related Cytokines

Communicating via tumour necrosis factor (TNF)-*α*, interleukin (IL)-1, IL-2, IL-12, and IFN-*γ* [19–21], T_H_1 cells play a fundamental role in acute rejection. These type 1 cytokines are upregulated early in the inflammatory process. After their release, IL-1*β* and TNF-*α* support the inflammatory response via activation of endothelial cells [22]. These cytokines act early in the inflammatory cascade, stimulating generation of cellular adhesion molecules, innate immune defence mechanisms, and participating in cross-talk between the various inflammatory pathways [23, 24]. IL-2 plays an essential role in resting T-cell activation and proliferation, contributing to T-cell maturation [25]. After T-cell induction via IL-2, IL-12 directs cellular maturation towards T_H_1, leading to a cell-mediated immune response [26]. IFN-*γ* influences both the innate and adaptive immune systems and is integral in the antigen presenting cell (APC) controlled balance between effector and suppressor T cells [27]. IFN-*γ* not only acts as the primary effector cytokine of IL-12 as part of cellular immunity, but also provides negative feedback control of IL-12 and indoleamine dioxygenase-mediated T-cell inhibition, under the control of APC's [27].

### 2.2. T_H_2-Cell-Related Cytokines

T_H_2 cell-related-cytokines include IL-4, IL-5, IL-10, and IL-13 [14, 28]. Type 2 cytokines are generally considered anti-inflammatory when associated with brain injury and BD, and in the early transplant period [29–31]. IL-4 inhibits formation of T_H_1 cells and encourages development of T_H_2 cells [29]. It also plays an essential role in B-cell generation of IgE [32]. IL-4 may activate macrophages via an alternative pathway that reduces inflammation through sequestration and metabolism of arginine, an essential requirement for nitric oxide generation by inflammatory IFN-*γ*-activated macrophages [33]. IL-4 has been postulated to depress T-cell activity through the production of indoleamine dioxygenase; Wang et al. demonstrated increased indoleamine dioxygenase produced by natural killer cells in IL-4-treated rat livers [33]. IL-13 is best known for its role in allergy. Through interaction with its receptor, IL-13 stimulates inflammatory cells as well as epithelial and smooth muscle cells [34]. This may contribute to smooth muscle hypertrophy and pulmonary hypertension in various lung diseases [34]. IL-13 inhibits cell-mediated immunity through downregulation of E-selectin, reduction of neutrophil recruitment, and macrophage inhibition [35]. IL-5 is essential for the development, recruitment and activation of eosinophils [36]. Once these cells are recruited to inflammatory sites, IL-5 is a potent costimulator of eosinophil degranulation and maintains their presence through inhibition of apoptosis [36, 37]. IL-5 also acts as a key mediator for generation of antigen-specific IgE. Furthermore, it is important for terminal B-cell differentiation, including the switch to mature IgM and IgG1 secreting plasma cells [38].

IL-10 acts to inhibit the production of inflammatory cytokines and upregulate inhibitors of IL-1 and TNF [15]. It may also specifically block the production of IL-1 and TNF [39]. Direct activity on inflammatory cells impairs or reverses the effects of proinflammatory mediators [39]. While IL-10 is classified as a type 2 cytokine, it is also able to be produced by T_H_1 cells under the influence of transforming growth factor (TGF)-*β* [19].

### 2.3. T_H_17-Cell-Related Cytokines

The T_H_17 cells are identified by their association with IL-6, IL-17, IL-21, IL-22, and IL-23 [14, 26, 40]. IL-17 and IL-23 direct T_H_17 cell differentiation, proliferation, and maturation [26]. Apart from directing T_H_17 development, IL-17 functions to stimulate production of chemokines, IL-1*β*, TNF-*α*, IL-6, and IL-8 [19, 41]. Its production is reinforced by IL-6, IL-23, and TGF-*β* [19, 41]. IL-8-related neutrophil attraction and activation may contribute partly to the inflammatory action of IL-17 [42]. IL-23 is an important upstream regulator of IL-17 expression [26, 43]. Generation of IL-17 by *γδ*-T cells is directly activated by IL-23, and these cells are an important source of IL-17 [43]. Furthermore, IL-23 induces IL-17 production from natural killer T cells [41]. IL-21 stimulates natural killer T cells, CD40 dependent B-cell proliferation, and T-cell expansion [44]. Hagn et al. recently demonstrated that incompletely activated  CD4^+^ T cells, through expression of IL-21 and CD40 ligand, stimulate B cells to differentiate into Granzyme B generating cytotoxic B cells [45].

IL-6 has been extensively investigated in many conditions. It's pro- and anti-inflammatory effects have recently been comprehensively reviewed [46]. Briefly, it is a pro-inflammatory agent which has been classified as a type 17 cytokine [47], although some authors may include it as a type 1 [48]. IL-6, the prototypical member of its family, acts through receptor complex formation with glycoprotein gp130 on the cell surface [46]. The IL-6 receptor molecule is present on the surface of hepatocytes, neutrophils, monocytes, and macrophages [46, 49]. Direct activation of these receptors is associated with an inflammatory response [49]. Other cells may also respond to IL-6 through a process termed *trans*-signalling [49]. Free soluble IL-6 receptor binds circulating IL-6 and then interacts with the ubiquitous cell surface protein, gp130, to affect cell signalling [46]. The dual roles of IL-6 may be partly explained by the differing signalling mechanisms. Soluble IL-6 receptor generated from apoptotic neutrophils in areas of inflammation activates signalling pathways after interaction with epithelial gp130, attracting regulatory monocytes and macrophages and contributing to resolution of inflammation [46].

### 2.4. T_reg_ Cells and Related Cytokines

Named due to their ability to downregulate inflammatory processes, T_reg_ cells are another important source of the anti-inflammatory IL-10. T_reg_ are closely related to T_H_17 cells; both lineages are derived from the same naïve T-cell precursor in a similar fashion to T_H_1/T_H_2 cells [14, 20]. Deknuydt et al. recently highlighted the fluidity of the T_H_17/T_reg_ balance by demonstrating that T_reg_ cells can be stimulated to become T_H_17 cells under the influence of IL-1*β* and IL-2 [14]. TGF-*β* also directs the differentiation of T-cell populations in inflammatory conditions and is important in the T_H_17/T_reg_ balance. TGF-*β* modulates the effects of IL-2, reducing expansion of inflammatory T-cell populations [19]. When acting synergistically with IL-2, TGF-*β* is able to direct naïve T cells to become T_reg_ cells [50]. Selective inhibition of T_H_1 producing mediators by TGF-*β* further contributes to the diversion from inflammatory T cells to T_reg_ cells, mediating the inflammatory response [19]. However, costimulation of TGF-*β* by IL-6 directs T-cell differentiation towards T_H_17 cells and production of type 17 cytokines [20]. T_reg_ cells are immunosuppressive through production of IL-10 and TGF-*β*, cellular anergy and direct contact with inflammatory cells [51]. 

## 3. Stage One of Potential Organ Injury: Brain Injury

Most brain dead donors suffer from three main causes of BD: cerebrovascular injury, anoxia, or traumatic brain injury (TBI) [52, 53]. Donor cause of death can significantly influence recipient survival rates, though this varies according to the organ. Renal transplant outcomes are adversely affected by cerebrovascular causes of BD [54, 55]. Lung transplant is unaffected by donor cause of death [56], while heart transplant outcomes remain controversial [57, 58]. For this reason, it is important to consider the pathophysiologic responses to severe central nervous system injury, and their systemic sequelae, prior to brain death.

### 3.1. Systemic Inflammatory Response Secondary to Brain Injury

Central nervous system (CNS) injury is associated with the systemic inflammatory response syndrome (SIRS). This can occur with an intact blood brain barrier (BBB), indicating an additional mechanism distinct from CNS-derived cytokine release [59, 60]. The link between the brain and SIRS has been comprehensively reviewed recently by Lu et al. [61]. Briefly, SIRS is associated with leukocyte mobilisation and recruitment to major organs, activation and release of inflammatory mediators, generation of ROS, increased vascular permeability, and organ dysfunction [62, 63]. Brain intraparenchymal injection of TNF-*α* recruits and activates systemic monocytes while IL-1*β* activates and recruits neutrophils via release of chemokines from the liver [59, 64].

TNF-*α* is released from the spleen in the early stages of brain injury to augment the peripheral inflammatory response [6, 65]. Lee et al. demonstrated upregulation of TNF-*α*, IL-1*β*, IL-4, and IL-6 in the spleens of rats with subarachnoid haemorrhage (SAH) [65]. Intravenous administration of neural stem cells attenuated the inflammatory response via a chaperone mechanism which was localised to the spleen and reversed on splenectomy [65]. Splenic inflammation may also be directly downregulated via vagal messages from the brain [6]. The SIRS response activates gut-derived inflammatory mediators, resulting in leaky gut wall [66]. This contributes to global inflammation through cytokine generation and systemic endotoxin exposure, worsening pulmonary inflammation and impairing oxygenation [66–68]. Similar to the spleen, gut generation of cytokines is also modulated by the CNS through vagal input [67]. 

### 3.2. Localised Response to Brain Injury and Loss of Blood Brain Barrier Function

Local responses to severe brain injury can be classified into two phases [69]. The primary phase is due to the insult itself and includes cellular death, direct BBB disruption, and cerebral oedema [69]. The secondary phase of injury is caused by elevated intracranial pressure (ICP), global brain ischaemia, excitotoxicity, metabolic derangements, and haemodynamic instability [69–71]. Whatever the cause of brain death, a cytokinaemia secondary to brain injury occurs prior to brain death itself [72–75].

Local inflammation, and the direct effect of the insult itself, causes the highly selective BBB to become disrupted [74, 76]. Matrix metalloproteinases (MMP), especially MMP-9, act to break down extracellular proteins, including basal lamina and endothelial tight junctions [77]. In a rat model of closed head trauma, Higashida et al. investigated the role of MMP-9 and hypoxia inducible factor (HIF) in cerebral oedema resulting from lost BBB integrity [77]. Inhibition of MMP-9 in this model significantly reduced the amount of brain oedema observed after 24 hours. Additionally, inhibition of HIF (an upstream regulator of protein expression associated with hypoxia) also significantly reduced the expression of MMP-9 and brain oedema [77]. This observation was confirmed in an intracranial haemorrhage model in rats; Wu et al. showed that MMP-9 is upregulated early after injury and is associated with brain oedema [78]. A postmortem study of intracranial haemorrhage confirmed these findings in humans [79].

The effect of the loss of BBB integrity is to allow bidirectional access of inflammatory cells and mediators [76, 80–84]. CNS-derived cytokines are then free to interact at receptors within the systemic tissues, inducing local inflammation and “priming” organs for further injury [81, 85]. The importance of brain injury-derived cytokinaemia was recently demonstrated by Graetz et al., who reviewed compartmental levels of IL-6 in SAH and found that elevated plasma IL-6 is associated with increased mortality [72]. This provides further evidence that isolated brain injury causes a systemic inflammatory response and upregulates the peripheral immune system [67, 83]. 

### 3.3. Type 1 Cytokines

Type 1 cytokines are upregulated in the brain after injury and contribute to BBB breakdown, vasospasm, and secondary injury [70, 86, 87]. The general roles of these and other inflammatory mediators have been previously reviewed [88]. Briefly, IL-1*β* is a pleiotropic proinflammatory mediator that stimulates multiple pathways of inflammation after brain injury [88]. TNF-*α* acts as a proinflammatory agent early in the inflammatory process in the CNS [89]. Microdialysis techniques have confirmed the presence of IL-1*β* and TNF-*α* in extracellular fluid after TBI and SAH [72, 87, 90]. Both of these cytokines are also released peripherally as part of a systemic acute phase response (APR) [6, 59, 91, 92]. IL-1*β* and TNF-*α* can be detected in blood within as little as one hour after brain ischaemia, even before significant neuronal cell death can be demonstrated [83, 93]. Quantitative systemic levels of type 1 cytokines may be affected by the type of brain insult; these were decreased in a middle cerebral artery occlusion model in mice, partially explaining the mechanism of the observed shift from T_H_1- to T_H_2-driven immunity after-stroke [16]. 

The soluble TNF Receptors (TNFR), p55 and p75, also contribute to the inflammatory process in traumatic brain injury, though the specifics of their involvement are not currently clear [84]. They act as anti-inflammatory agents through free TNF scavenging, although TNFR levels are more closely correlated to mortality in potential donors than TNF itself [84, 89]. This observation may actually reflect an imbalance in pro- and anti-inflammatory mechanisms or simply be due to the very short half-life of TNF [84]. 

### 3.4. Type 2 Cytokines

A recent study of stroke in IL-4 knockout mice showed that IL-4 reduces the T_H_1 : T_H_2 cell ratio and infarct volume, and improves neurological outcome [29]. Studies in humans have shown that brain-derived IL-4 can be detected in the jugular vein in patients with serious head injury [94]. A post-mortem study of TBI patients confirmed elevated IL-4 in brain tissue [70]. IL-13 has been less studied in brain injury. One *in vitro* study of IL-13 and IL-4 did show that these mediators induced apoptosis of activated microglia, which may account for part of the observed anti-inflammatory effect [95]. IL-13 is not significantly elevated in the plasma after TBI [31].

IL-10, a type 2 cytokine with anti-inflammatory properties [29, 30], plays a protective role in the CNS, reducing infarct size in stroke patients [39, 96]. Analysis of post-mortem TBI brains confirmed the presence of IL-10, though levels were more modest than similarly identified pro-inflammatory cytokines [70]. This was consistent with intraparenchymal levels measured by microdialysis in TBI and SAH patients [90]. Overflow of IL-10 into the cerebrospinal fluid (CSF) after TBI has also been demonstrated [97]. Systemic IL-10 levels peak early in TBI patients, declining to baseline within 48 hours [84]. Although IL-10 decreases inflammation through its immunomodulatory action, it also increases susceptibility to infection through immune system downregulation [96]. 

### 3.5. Type 17 Cytokines

In the CNS, IL-6 plays a dichotomous role through modulation of glial responses and neuronal survival, contributing to the early inflammatory response, but modulating later inflammatory pathways to assist with brain recovery [89, 98–100]. While its proinflammatory role is well known, it has also been shown to protect against excitotoxicity *in vitro* and brain ischaemic or excitotoxic states *in vivo* [98]. The specifics of how this balance are achieved are less clear [98]. One suggestion is that the role of IL-6 depends on the amount of neuronal cell damage and is concentration dependent, but it is also probably subject to negative feedback inhibition via crosstalk between NMDA and IL-6 receptors [98]. It may also downregulate inflammation through stimulating IL-1 receptor antagonist [90]. Microdialysis techniques have confirmed that IL-6 is acutely increased after brain injury [72, 73]. Furthermore, Graetz et al. demonstrated that IL-6 is released from the brain parenchyma into the systemic circulation after brain injury, particularly in the presence of high ICP [72]. Previous studies have also shown that IL-6 interferes with BBB integrity [72, 80]. Similar to IL-4, IL-6 is detectable in jugular blood, and the transcranial gradient correlates with poor outcome in TBI [72, 80]. The APR is stimulated by circulating IL-6 [101] and this may provide a link between central injury and the peripheral immune response seen with intracranial injury [83]. 

The roles of IL-17 and IL-23 in acute brain injury remain to be fully elucidated. While a role has been established in central autoimmune disorders including experimental models of multiple sclerosis [13], less has been published on acute CNS injury. Murine models demonstrate that both of these interleukins are locally upregulated after stroke [102–104]. Currently, there are no published data on their peripheral release after acute brain injury.

### 3.6. The Endothelin Axis in Brain Injury

Endothelin-1 (ET-1) is the most active member of a family of small polypeptides which are potent vasoconstrictors, mitogens of smooth muscle cells, and stimulators of fibroblasts (Table 1) [105–109]. ET-1 is an important mediator in TBI, stroke, and SAH [110–113]. In acute brain injury, ET-1 leads to constriction of large vessels, altering the normal balance between vascular relaxation and constriction, resulting in impaired cerebral blood flow [113]. This alteration of blood flow has been targeted in studies of SAH [114]. Clazosentan, an ET receptor A antagonist, reduces large cerebral artery vasospasm in murine models, but this did not reduce other mechanisms of secondary brain injury [114]. Salonia et al. analysed CSF levels of ET-1 in paediatric head trauma [113]. They found that ET-1 is significantly elevated after injury and remains so for up to 5 days. Central production of ET-1 in adult TBI was confirmed by Chatfield et al. [111]. Their analysis of the juguloarterial gradient showed that ET-1 is produced intracranially and spills over into the systemic circulation.

## 4. Stage Two of Potential Organ Injury: Brain Death

**Figure 2:**
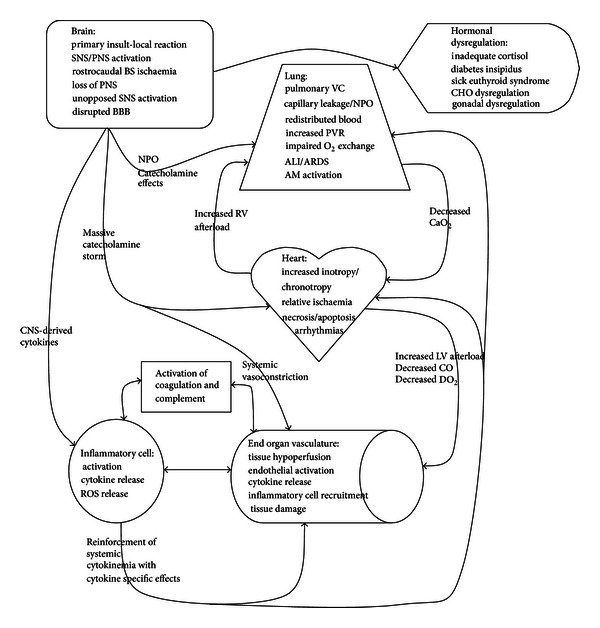
Interaction of homeostatic mechanisms after BD. ALI: acute lung injury,   AM: alveolar macrophage, ARDS: acute respiratory distress syndrome, CaO_2_: arterial oxygen content, BBB: blood brain barrier, BS: brain stem, CHO: carbohydrate, CNS: central nervous system, CO: cardiac output, DO_2_: oxygen delivery, NPO: neurogenic pulmonary oedema, PNS: parasympathetic nervous system, PVR: pulmonary vascular resistance, ROS: reactive oxygen species, VC: vasoconstriction.

Serious brain injury, augmented by local inflammation, may eventually lead to an irretrievable state of impaired brain function and brain death. BD then further causes a massive autonomic storm and cytokinaemia which increases the inflammatory state of the individual [126, 140, 141]. A complex interplay of immunologic [142], coagulopathic [143], autonomic, haemodynamic, and endocrine [144, 145] dysregulation drives inflammation through local and global cytokine release, cellular activation, organ priming, IRI, and secondary ischaemic insult (Figure 2).

### 4.1. The Autonomic Nervous System during Brain Death

Brain stem dysfunction is associated with extreme physiological perturbations due to its “master control” function [146–148]. Brain stem failure secondary to high ICP occurs in a rostrocaudal direction, with initial hypertension and bradycardia (classically known as Cushing's reflex [149, 150]), followed by an intense “sympathetic storm” which remains unopposed due to ischaemia of the parasympathetic vagal nucleus [151, 152]. This storm results from an overwhelming release of catecholamines in an attempt to perfuse the brain by increasing the mean arterial pressure (MAP) to overcome the elevated ICP [153, 154]. Such changes in autonomic outflow can be detected prior to the occurrence of brain death [155]. The initial massive upsurge in sympathetic tone results in widespread vasoconstriction and microthrombus formation, impairing organ and tissue perfusion [146].

As the ICP outpaces the MAP, ischaemia progresses down the brain stem, sympathetic centres become necrotic, vascular and myocardial sympathetic stimulation drops and a second phase of hypotension ensues [146, 156, 157]. The resulting uncontrolled hypotension further impairs the already tenuous end organ perfusion that resulted during the sympathetic storm [156].

While the effects of the sympathetic nervous system are the most obvious clinically during and after BD, inflammatory and haemodynamic responses are also influenced by the parasympathetic nervous system (PNS). The effect of BD is to inhibit PNS-mediated anti-inflammatory responses by direct destruction of vagal centres in the brain stem [67]. Under normal conditions, vagal stimulation directly decreases inflammation via cholinergic receptors on inflammatory cells [158, 159]. Central activation of vagal efferent pathways downregulates inflammation in the brain, gut, and spleen [6, 67]. Balance is normally achieved through negative feedback by the innate immune system interacting with the PNS via IL-1 receptors in the parasympathetic paraganglia [67].

### 4.2. Cytokine Upregulation after Brain Death

#### 4.2.1. Type 1-Associated Cytokines

Cytokine upregulation after BD has been recognised for many years [66, 160]. Animal models have shown that serum levels of IL-1*β* and TNF-*α* may be influenced by the rate of induction of brain death [22, 161]. Avlonitis et al. reported that explosive brain death induced a rapid increase in IL-1*β*, with significantly elevated levels detectable within one hour, remaining so throughout the duration of the study [161]. TNF-*α* levels initially rose and then decreased by five hours, though it remained above baseline [161]. Zhu et al. showed that gradual induction of brain death leads to steady elevation of IL-1*β* over 24 hours in a pig model [162]. Conversely, Damman and colleagues, utilising gradual BD induction in a rat model, showed that IL-1*β* and TNF-*α* did not change significantly over the four hours of their study [101]. Interestingly, this group also analysed serum cytokine levels in human BD donors and showed that they were not significantly elevated [101]. Cypel and colleagues recently reported that TNF-*α* and IL-1*β* mRNA are significantly elevated in lungs rejected for transplant, highlighting the clinical importance of these proinflammatory cytokines [163].

#### 4.2.2. Type 2-Associated Cytokines Including IL-10

Early studies of cytokine upregulation after BD suggested that type 2 cytokines are not significant contributors to BD-induced inflammation [160]. Takada et al. did not show upregulation of IL-4 in rat kidneys, hearts, livers, or lungs after BD [160]. Weiss et al. studied cytokine expression at various timepoints during the liver transplantation process [91]. This group reported that IL-4 expression is increased after brain death [91]. IL-10 is elevated in the plasma of human BD donors [66, 123, 126]. Additionally, IL-10 has been shown to be upregulated in human livers [91] and kidneys [164]. Work undertaken by Li et al. suggested that IL-10 expression after BD may be important in stimulating apoptosis of graft infiltrating lymphocytes through activation of the Fas/Fas Ligand pathway [165]. There is little published in the literature investigating the role of IL-5 and IL-13 during brain death. This may be an area for future research.

#### 4.2.3. Type 17-Associated Cytokines

IL-6 is heavily implicated in BD-related inflammation [127, 128], where it is an important instigator of the generalised APR [101]. The levels increase in human brain dead donors up until the time of organ retrieval [101]. Systemic venous and CNS-derived IL-6 is significantly higher at brain death than at admission to the intensive care unit (ICU) in TBI patients that progress to BD [80]. Brain death induces the production of IL-6 in multiple organs, including the kidney [166], heart [167], liver [168], and lung [141]. IL-6 signalling induces nitric oxide synthase in cardiac myocytes [167] and contributes to early haemodynamic compromise in the donor via direct negative inotropy [121, 127]. IL-6 mRNA and protein are elevated in nonstructural donor heart dysfunction [167].

Damman et al. recently investigated IL-6-related renal acute phase protein synthesis in rats [101]. As expected, IL-6 was upregulated after brain death. This correlated with an increase in renal acute phase proteins, notably complement 3 (C3), fibrinogen, *α*2-macroglobulin, and haptoglobin [101]. Furthermore, *in vitro* analysis indicated that renal production of C3 is directly related to IL-6 exposure [101].

Overall, elevated plasma levels of IL-6 are associated with poorer transplantation outcomes [91, 168]. Murugan and colleagues demonstrated an inverse relationship between donor plasma IL-6 levels and recipient six-month hospital-free survival [126]. Kaneda et al. also showed that higher donor IL-6 levels increased the risk of recipient death within 30 days of lung transplant [169].

T_H_17 cells, through production of IL-17, stimulate inflammation in donor organs [170]. Pretransplant renal biopsies from deceased donors showed little elevation of IL-17 positive cells, though few graft infiltrating cells were demonstrated in the biopsy samples [170]. Although a number of authors have studied IL-17 in the context of chronic rejection, the role of BD donor IL-17 currently remains unexplored.

#### 4.2.4. T_reg_-Associated Cytokines

TGF-*β* is upregulated in heart and lung tissue in animal models [81]. Elevated TGF-*β* mRNA has been identified in renal and liver biopsies from brain dead donors [91, 164]. Weiss et al. showed that the greatest stimulus for TGF-*β* expression in liver grafts is BD itself [91]. A slight decrease in expression occurred prior to cold storage and to reperfusion. TGF-*β* mRNA expression increased by one hour after implantation and reperfusion but did not exceed levels measured before surgical manipulation (i.e., after BD alone) [91]. Skrabal et al. also demonstrated that TGF-*β* mRNA transcription is increased in donor heart and lungs in a porcine model of brain death [81]. The role of TGF-*β* in acute organ injury may relate to its role in the T_H_17/T_reg_ balance [20, 50]; however, increased expression prior to transplantation may start fibrotic processes through activation of MMP's and tissue inhibitor of metalloproteinases (TIMP's). MMP-2, -9, TIMP-1, and -2 expression is increased after BD in pulmonary tissue [115]. 

#### 4.2.5. Interleukin 8

IL-8 is a chemokine which attracts and activates neutrophils [132, 171]. Similar to other cytokines, IL-8 is produced peripherally after BD where it stimulates neutrophil-driven angiogenesis and fibroproliferation [132, 172]. IL-8-induced neovascularisation, alveolar-capillary disruption, and extracellular matrix deposition contribute to the development of acute lung injury after brain death [132]. In lung donors, bronchoalveolar lavage fluid IL-8 levels are positively correlated with neutrophil infiltration in pretransplant lung tissue, contributing to early graft dysfunction [132]. 

#### 4.2.6. The Endothelin Axis

ET-1 release from endothelium is stimulated by noradrenaline, thrombin, and TGF-*β* [139]. Animal experiments have shown that ET-1 is upregulated in serum and in donor lung after BD and that this is related to MMP activation [115, 173]. Salama et al. demonstrated a correlation between donor ET-1 and primary graft dysfunction (PGD) [174]. In this study, ET-1 upregulation (as measured by donor lung mRNA and donor serum levels) adversely affected recipients after transplantation, contributing to the development of PGD.

## 5. Stage Three of Potential Organ Injury: Ischaemia Reperfusion Injury

Ischaemia reperfusion injury is implicated in early and late stage transplant complications [175]. IRI leads to organ dysfunction through induction of cytokines, generation of free radicals, and activation of immunocompetent cells [175, 176]. Endothelial cell dysfunction secondary to IRI is a key contributor to chronic allograft dysfunction in hearts [177], lungs [11], livers [178], and kidneys [179]. Early injury to cells occurs as a direct result of ischaemia, with impaired oxygen delivery, altered energy metabolism, and accumulation of waste products. Cell death occurs through necrosis and apoptosis, the latter through caspase signalling [66, 180]. Further injury occurs upon reperfusion, with recruitment of inflammatory cells, interaction between local and systemic cytokine signalling systems, and generation of ROS [127, 181, 182]. 

APC's of the innate immune system play a key role providing antigens and costimulatory molecules to activate the adaptive immune system, contributing to IRI and early graft dysfunction. Activation of cellular immunity can be classified as direct or indirect [181]. Direct activation occurs due to the transfer of donor APC's in the allograft, which activate recipient T_H_1 cells [181]. Atkinson et al. recently demonstrated that passenger leukocytes are recruited to donor hearts after BD in a murine model [22]. This finding was also confirmed in lung [183] and renal allografts [184]. Gelman et al. also demonstrated that recipient T cells interact with donor APC's and that this is sufficient to activate an inflammatory response [183]. Alternatively, the indirect pathway results from the interaction of recipient APC's with native T cells to stimulate inflammation. 

### 5.1. Contribution of Preservation Strategies to Cytokine Expression

Hypothermic preservation strategies are widely used to decrease inflammation, depress the metabolic rate of cells, and reduce the effects of ischaemia [185]. However, cold storage does cause cell death via both apoptosis and necrosis [15]. BD donor organs predominantly display the latter mechanism [178]. The duration and type (warm or cold) of ischaemic time may also directly influence cytokine production. A correlation was recently identified between cold ischaemia time and levels of IL-1 and IL-8 in human liver transplants [186]. Warm ischaemia time correlated with IL-6 and IL-10 in the same study. Significantly, the authors found that the excess cytokines generated by hepatic graft warm ischaemia time resulted in systemic adverse effects, most notably increased intraoperative pulmonary shunt [186]. Another study found that, while cold ischaemic time per se did not adversely affect liver function, the associated graft-generated IL-8 did correlate with PGD [187]. Weiss et al., in a study of transplanted human livers, showed that IL-4 was increased in BD donors prior to explantation, but cold ischaemia and reperfusion did not result in further increases in the cytokine [91]. Indeed, while it was elevated compared to living donors, it failed to reach statistical significance at time points other than immediately after laparotomy. IL-10 was highly expressed prior to organ preservation, but cold ischaemia and reperfusion did not result in further elevation of this cytokine [91]. Livers from living donors showed a relatively greater increase in expression of IL-10 one hour after reperfusion than BD organs, which may partially contribute to better outcomes with organs from these donors [91]. 

Delayed graft function in transplanted kidneys has been shown to be dependent on cold ischaemic time [170]. Kaminska et al. showed that while cytokine upregulation occurred, associated with brain death, mRNA expression did not increase further after cold ischaemia and prior to reperfusion [164]. In keeping with this, de Vries et al. were unable to detect an arteriovenous difference across human BD donor kidneys for multiple cytokines, including IL-4, IL-5, IL-10, and IL-13 [184]. Cold ischaemia and reperfusion do not induce excess TGF-*β* mRNA production, indicating that the primary stimulus for this mediator is brain death itself [164]. 

### 5.2. Other Mediators of Ischaemia Reperfusion Injury

The combination of BD and IRI activates allografts greater than either insult alone. Kusaka et al. studied rat renal isografts to analyse gene activation after BD, IRI, or combined BD/IRI [175]. They found that BD primarily upregulated cytokines, chemokines and adhesion molecules while IRI tended to upregulate transcription factors. Combined BD/IRI was synergistic in enhancing upregulation of these genes. More recent work has maintained these findings. Inhibition of JNK, a phosphorylator of the transcription factor c-Jun, decreases IRI-induced renal damage in rats [179]. In humans, de Vries et al. demonstrated that reperfusion of BD kidneys generates higher cytokine levels than living donor allografts (i.e., those that only underwent IRI) [184]. 

Complement interacts with, and reinforces, the inflammatory process of IRI by increasing TNF-*α* and IL-1 [22]. C3a and C5a, potent anaphylatoxins generated by the complement cascade, activate mast cells and neutrophils [182]. While the specific mechanism of complement activation in BD is unknown, it is postulated that ischaemia leads to defects in cell membranes, uncovering neoepitopes via exposure of internal cellular components to the humoral immune system, which leads to interaction with natural IgM and activation of the classical complement pathway [182]. ROS generated during infarction and IRI may lead to lipid peroxidation and alteration of cellular cytoskeletal structure providing further neoepitopes for IgM [182]. 

The importance of toll-like receptors (TLR) in IRI is currently being investigated. It was previously noted that low levels of lipopolysaccharide (LPS) may precondition and therefore protect the lung from IRI [188]. Merry et al. demonstrated that low-dose preconditioning with LPS in rat lung ischaemia reduced injury [188]. The authors postulated that this may be due to LPS activating TLR-4 via an alternative pathway that results in protective interferon and IL-10 generation. Unfortunately, they did not measure IL-10 protein or mRNA to confirm this hypothesis. The role of TLR's in renal IRI has recently been reviewed elsewhere [12]. TLRs may also contribute to inflammation through interaction with T cells via cytokine signalling. APC TLR activation leads to generation of cytokines, including IL-6, which may decrease the sensitivity of T_H_1 cells to the immunosuppressive effects of T_reg_ cells [189]. Additionally, TLR on T_reg_ cells may directly inhibit their immunosuppressive effects [189].

ET-1 contributes to IRI through activated neutrophils, leading to endothelial injury, neutrophil superoxide production and generation of ROS [115]. Both ET-1 and its receptors are upregulated in the lungs after brain death [115]. Alveolar macrophages have been demonstrated to increase expression of endothelin receptors in the donor lung in animal models [115]. This may then prime passenger macrophages for further activation by recipient ET-1, which is generated in the pro-inflammatory environment of chronic lung disease, surgery, and the posttransplant course [115, 190].

Heme-oxygenase-1 (HO-1) is essential for the metabolism of heme to carbon monoxide, free iron, and biliverdin [25]. Its ability to reduce injury secondary to IRI, with resulting better recipient outcomes after transplantation, has been the subject of much research. HO-1 exerts its beneficial effects through antioxidant, antiapoptotic, and anti-inflammatory mechanisms [25, 191–193]. Carbon monoxide contributes to these beneficial effects through inhibiting T-cell proliferation and IL-2 secretion [25]. Zhou et al., in studying a rat model of BD, demonstrated improved lung function and decreased lung injury when carbon monoxide was administered at 250 ppm [194]. Carbon monoxide decreased myeloperoxidase activity, TNF-*α*, and IL-6 [194]. More recently, the same group demonstrated that both carbon monoxide and biliverdin reduce myeloperoxidase activity and cytokine signalling while improving respiratory mechanics in rat lung after BD [195]. HO-1 has also been linked to anti-inflammatory cytokine generation. IL-10 production secondary to HO-1 is increased in both BD [192] and non-BD models [196]. HO-1 may also be an important mediator of IL-13's anti-inflammatory effect [191, 197].

## 6. Management Implications and Potential Future Directions

Recipients of organs from brain dead donors continue to have poorer outcomes than those that receive living donor organs. Aggressive donor management (ADM) improves both quality and quantity of organs available for transplant [198]. Current ADM recommendations include early identification of potential donors, ICU admission, pulmonary artery catheterisation, aggressive fluid management, vasopressors, hormonal resuscitation therapy, pulmonary toilet, and bronchoscopy [148, 199–202]. 

Even with ADM, up to 25% of potential donors are lost due to haemodynamic instability [148]. Studies comparing use of noradrenaline and vasopressin as pressor agents demonstrate differing effects on transplantable organs. Although no studies directly comparing these agents in humans have been published, animal models suggest that both agents decrease lung inflammation and serum cytokine release [68]. While a similar effect is seen in the kidney, hepatic inflammation is increased by both agents [203]. Dopamine decreased monocyte kidney graft infiltration and markers of inflammation in a rat model of BD [193]. Additionally, dopamine increased the expression of HO-1 [193]. Regardless of the agents used, the evidence supports that aggressive haemodynamic monitoring and management do convert marginal donors to acceptable donors [141, 145]. 

The inflammatory cascade may be downregulated by ADM. Currently, there are no standard interventions specifically directed at individual cytokines, though many are being investigated (Table 2). Steroid administration, as part of hormonal resuscitation, is now commonplace in the management of organ donors and, in addition to addressing a relatively inadequate adrenal response, reduces inflammatory cytokines to levels similar to living donors [141, 151, 168]. 

Other methods directly addressing anti-inflammatory mechanisms are currently being investigated. Gene transfer of IL-10 holds great promise. Manning and colleagues investigated viral IL-10 (virIL-10) transfer into a rat model of lung IRI using mesenchymal stem cells [11]. This study showed that virIL-10 was detectable in the lungs and that presence of this cytokine was related to improved lung function, less microscopic pathology, and decreased lung oedema at four hours after injury. Gene transfer pretreatment of rat liver grafts to generate recombinant human IL-10 significantly decreases IRI and markers of apoptosis, with upregulation of HO-1 and the antiapoptotic agent, Bcl-2 [15]. HO-1 may then act as a downstream regulator of protective mechanisms in IRI [15]. 

HO-1 or its metabolites (carbon monoxide and biliverdin) may offer potential therapeutic benefits [25, 192, 194, 195]. Overexpression of HO-1, through adeno-associated virus gene transfer, was associated with a beneficial increase in HO-1 expression [192]. This resulted in downregulation of IL-2 and TNF-*α*, decreased infiltration of cytotoxic and helper T-cells, and an increase in IL-10, TGF-*β*, and T_reg_ infiltration in transplanted rat livers [192]. IL-13 gene transfer in rat livers increased HO-1 expression with reduced evidence of IRI [197]. Inhibition of HO-1 activity reversed this effect, suggesting that part of IL-13's anti-inflammatory properties in IRI is mediated by HO-1 [197].

Hypothermic ischaemic storage prior to transplantation does not allow sufficient metabolic activity for gene transfer to be beneficial [204]. Cypel et al. therefore trialled an *ex vivo* lung perfusion (EVLP) model to transfer recombinant human IL-10 genes into porcine lungs [204]. Perfusate IL-10 was increased while IL-6 decreased. This effect was maintained after transplantation and four hours of reperfusion. Lung function, as assessed by P_a_O_2_ : F_i_O_2_ ratio, was significantly improved in the transfected lungs. When transfection was trialled in human lungs rejected for transplantation, Cypel and colleagues found similar results including improved gas exchange and pulmonary vascular resistance [204].

Lung conditioning using EVLP is able to improve the function of lungs initially rejected for transplant [205]. Sadaria et al. have established a baseline cytokine profile of human lungs undergoing EVLP [205]. Cytokine analysis during 12 hours of EVLP showed an upregulation in IL-6, IL-8, G-CSF, and MCP-1 [205]. IL-1*β*, IL-4, IL-7, IL-12, and TNF-*α* were detectable but remained unchanged [205]. IL-17 was undetectable, as were IL-10 and IL-13 [205]. Kakishita et al. also investigated the cytokine profile of EVLP in pigs [206]. Inflammatory cytokines were similarly elevated. Interestingly, based on a previously published concept of haemoadsorption of cytokines [123], Kakishita investigated the benefit removing perfusate cytokines within the circuit. Cytokine levels were significantly reduced with haemoadsorption, but oxygenation, pulmonary vascular resistance, peak airway pressure, and myeloperoxidase activity (as a marker of neutrophil accumulation) were not statistically different [206].

Numerous other agents have been investigated as part of organ protection and preservation strategies. Donor simvastatin may reduce IRI in cardiac allografts [177]. This agent appears to work through multiple mechanisms and provides a lasting effect after a single dose to the donor prior to graft removal [177]. Organ donors in this animal model were not brain dead; therefore, simvastatin's effects seem to be related to downregulation of ischaemia reperfusion injury. A study of N-acetylcysteine after pig non-BD lung transplantation demonstrated increased glutathione and downregulation of the inflammatory transcription factor NF*κ*B in tissue samples [171]. IL-6 and IL-8 levels were also reduced. Lung function was improved despite extended cold ischaemia and reperfusion [171]. Intraoperative administration of N-acetylcysteine to human liver transplant recipients significantly increased the transhepatic gradient of IL-4 and IL-10 around the time of reperfusion, but not at other measured time points [207]. The authors theorised that the presence of these anti-inflammatory cytokines at reperfusion may benefit recipients through downregulation of inflammation. Unfortunately, although the agent was administered as a continuous infusion for 24 hours, no further information is given about levels of cytokines later than the first hour of transplant, nor any information about hepatic biochemistry and patient outcomes. 

In renal transplantation, carbamylated erythropoietin (EPO) downregulated renal IL-1*β* and IL-6 in a rat model of brain death [166]. This agent retains the protective effects of EPO without stimulating haematopoiesis [166]. Utilising an isolated perfused kidney circuit, Nijboer and colleagues demonstrated that carbamylated EPO downregulated IL-1*β* and IL-6, reduced neutrophil infiltration, and reversed brain death-induced renal impairment. Of note, other authors are also investigating EPO in preventing brain IRI [208]. Such use in pre-BD conditions may eventually spill over to benefit the recipients of organs from these patients in the case of nonsurvival.

Further research is required into the impact of pre-BD management of organ donors. There are substantial data examining the management of TBI or SAH patients which specifically addresses inflammatory/anti-inflammatory interventions and long-term recovery. The impact of such management on the transplanted organs of those that fail treatment and become BD organ donors may reveal interesting results. 

## 7. Conclusion

Engrafted organs undergo significant pathophysiological challenges as they are transplanted from the donor to the recipient. Brain injury, brain death, ischaemia, and reperfusion all contribute to inflammation and injury. As has been discussed, a vast amount of research is ongoing at each of these steps of transplant. Understanding the molecular inflammatory responses and utilising interventions that can reduce haemodynamic instability, inflammation, and IRI is the key to further advancing donor management. With time and more successful interventions, it may be possible to further address the ongoing shortage of donor organs and decrease the number of patients who die whilst waiting for a transplant.

## Figures and Tables

**Table 1:** Properties of endothelin.

Endothelin	
Endothelin subtypes [105, 106, 115]	
ET-1 ET-2 ET-3	
Sites of production [105]	
Smooth muscle cells Cardiomyocytes Leukocytes Macrophages Mesangial cells Airway epithelium Alveolar epithelial cells	
Receptor subtypes [105, 107, 108, 116, 117]	
Endothelin receptor A Endothelin receptor B	
Action	
Endothelin receptor A [105, 107, 108, 116, 117] Smooth muscle contraction Fibrogenesis Endothelin receptor B [105, 107, 108, 116, 117] Smooth muscle contraction Smooth muscle relaxation ET-1 clearance	
Localisation of receptors [105, 107, 115]	
Heart Endocardium Conducting system Coronary vessels Lung Kidneys CNS Liver Neutrophils	
Stimulators of release [105]	
Endothelial shear stress Thrombin AT2 Cytokines Free radicals Catecholamines	
Inhibitors of release [105]	
NO ANP Prostacyclin	

ET: endothelin, AT2: angiotensin 2, NO: nitric oxide, ANP: atrial natriuretic peptide, CNS: central nervous system.

**Table 2:** Major cytokines associated with brain injury and BD.

Cytokine/chemokine	Organs/cells upregulated in BD/CNS injury	Stimulation in BD/TBI	Action	Potential therapeutic agents in brain injury
TNF-*α*	CNS—astrocytes, microglia, and neurons [70, 118] Endothelial cells [118] Lungs [119] Splenocytes, macrophages [6]	Infection, TBI, SAH [118]	Endothelial cell detachment/apoptosis, activation caspase-3, disruption of BBB [118] Induction of CAM's, and other inflammatory cytokines [120] Impairment of cardiac function [121]	IFN-*β* [119], NNZ-2566 [122], etanercept, and IFN inhibitors [60] Haemoadsorption [123]

IL-1*β*	CNS—neurons, microglia, and infiltrating macrophages [124] Endothelial cells [118]	Neuroexcitation, infection, and trauma [124] SAH [118]	Synaptic modulation, central regulation of systemic inflammatory response [124] Proinflammatory, activation of NF*κ*B and SAPK with upregulation of E-selectin/ICAM/VCAM [118]	IL-1RA [124] and NNZ-2566 [122] Haemoadsorption [123]

IL-6	CNS—microglia [125] Kidney, liver, spleen, and heart [80, 121, 126–128] Macrophages [121]	IL-1*β* [118] TNF-*α* [121] Sepsis, major surgery, heart failure, multitrauma, and burns [80, 84, 127, 129, 130]	Regulator of inflammation—inhibition of TNF and upregulation of control of glial responses and neuronal survival [98–100] IL-1RA in CNS, induction of NGF [122] Disruption of BBB [118] Inducer of acute phase reaction [70, 131] Cardiac dysfunction, fibroblast activation [127]	Haemoadsorption [123]

IL-8/CXCL-8/MIP-2	Microglia [125] Lung—alveolar macrophages, endothelial cells [132, 133]	Trauma, ischaemia, SAH, ET-1 [118] TNF-*α*, IL-1*β* [120]	Disruption of BBB [118, 120] CXC chemokine—neutrophil migration and activation [120] Induces ROS by neutrophils [131]	Haemoadsorption [123]

IL-10	Macrophages, microglia [125] Splenocytes [83]	TBI [97] Burns, MT, surgery, and infection [131]	Anti-inflammatory—downregulates TNF-*α*, IL-1*β*, and IFN-*γ*, upregulates antagonists [39, 134] Reverses effect of proinflammatory cytokines directly on cells [39]	Haemoadsorption [123]

E-Selectin	Endothelial cells in multiple organs [119]	IL-1*β* [118] TNF-*α* [121] TBI [122] SAH [118]	Essential for neutrophil rolling, margination, and diapedesis [118]	

ICAM	Endothelial cells in multiple organs [119]	IL-1*β* [118] TNF-*α* [121] SAH [118]	Essential for neutrophil rolling, margination, and diapedesis [118]	Monoclonal antibodies [118] and IFN-*β* [119]

VCAM	Endothelial cells in multiple organs [119]	IL-1*β* [118] TNF-*α* [121] SAH [118]	Essential for neutrophil rolling, margination, and diapedesis [118]	Monoclonal antibodies [118] and IFN-*β* [119]

TGF-*β*	Macrophages, microglia, astrocytes, and neurons [135, 136] Platelets, choroid epithelium [137]	Constitutively expressed by microglia [134] SAH [137]	Anti-inflammatory, may block activation by IL-1*β* [125] Regulates T-cell survival and function [136] Suppresses IFN-*γ*-induced macrophage upregulation, cytokine and chemokine generation [136] Downregulation of adhesion molecules [136] Reduces COX-2 production in microglia [125] ECM component generation [138] Angiogenesis [137] ET-1 generation [139]	Haemoadsorption [123]

IFN-*γ*	Microglia [125] Macrophages [122]	TBI, SAH [122, 125]	Upregulation of CAM's, chemokines, and innate immune system cells [122]	IFN inhibitors [60]

COX-2	CNS—Microglia, endothelial cells [125]	Inflammatory mediators including IL-1*β*, TNF-*α*, and IL-6 [125]	Production of prostaglandins, reinforcement of inflammation [125]	COX inhibitors [125]

BD: brain stem death, TBI: traumatic brain injury, CNS: central nervous system, SAH: subarachnoid haemorrhage, BBB: blood brain barrier, MT: multitrauma, ECM: extracellular matrix, COX: cyclooxygenase, IL: interleukin, TNF: tumour necrosis factor, CAM: cellular adhesion molecule (ICAM: intercellular adhesion molecule/VCAM: vascular cellular adhesion molecule), NF_*κ*_B: nuclear factor *κ* B, SAPK: stress-activated protein kinases, MIP-2: macrophage inflammatory protein 2, TGF: transforming growth factor, IFN: interferon, NGF: nerve growth factor.

## Abbreviations

ADM: Aggressive donor management
APC: Antigen presenting cell
APR: Acute phase response
BBB: Blood brain barrier
BD: Brain death
C: Complement
CD: Cluster of differentiation
CNS: Central nervous system
CSF: Cerebrospinal fluid
EPO: Erythropoietin
ET: Endothelin
EVLP: *Ex vivo *lung perfusion
G-CSF: Granulocyte colony stimulating factor
HIF: Hypoxia inducible factor
HO: Heme oxygenase
ICP: Intracranial pressure
ICU: Intensive care unit
IFN: Interferon
IL: Interleukin
IRI: Ischaemia reperfusion injury
LPS: Lipopolysaccharide
MAP: Mean arterial pressure
MCP: Monocyte chemoattractant protein
MMP: Matrix metalloproteinase
mRNA: Messenger ribonucleic acid
NFKB: Nuclear factor kappa B
NMDA: N-Methyl D-aspartic acid
PGD: Primary graft dysfunction
PNS: Parasympathetic nervous system
ROS: Reactive oxygen species
SAH: Subarachnoid haemorrhage
SIRS: Systemic inflammatory response syndrome
SNS: Sympathetic nervous system
TBI: Traumatic brain injury
TGF: Transforming growth factor
TH: Helper T cell
TIMP: Tissue inhibitor of metalloproteinases
TLR: Toll-like receptor
TNF: Tumour necrosis factor
TNFR: Tumour necrosis factor receptor
T_reg_: Regulatory T cell.
